# Supplementary material for: The interactome of intact mitochondria by cross-linking mass spectrometry provides evidence for coexisting respiratory supercomplexes
Source: Mol Cell Proteomics. 2017 Dec 8;17(2):216–32. doi: 10.1074/mcp.RA117.000470 (PMC5795388; doi:10.1074/mcp.RA117.000470)
Supplement: Supplemental Data [file supp_RA117.000470_133922_0_supp_23978_fzffwf.zip › spectra_annotation/mito_spectra_annotation/10-1-5-1-11-1.pdf]

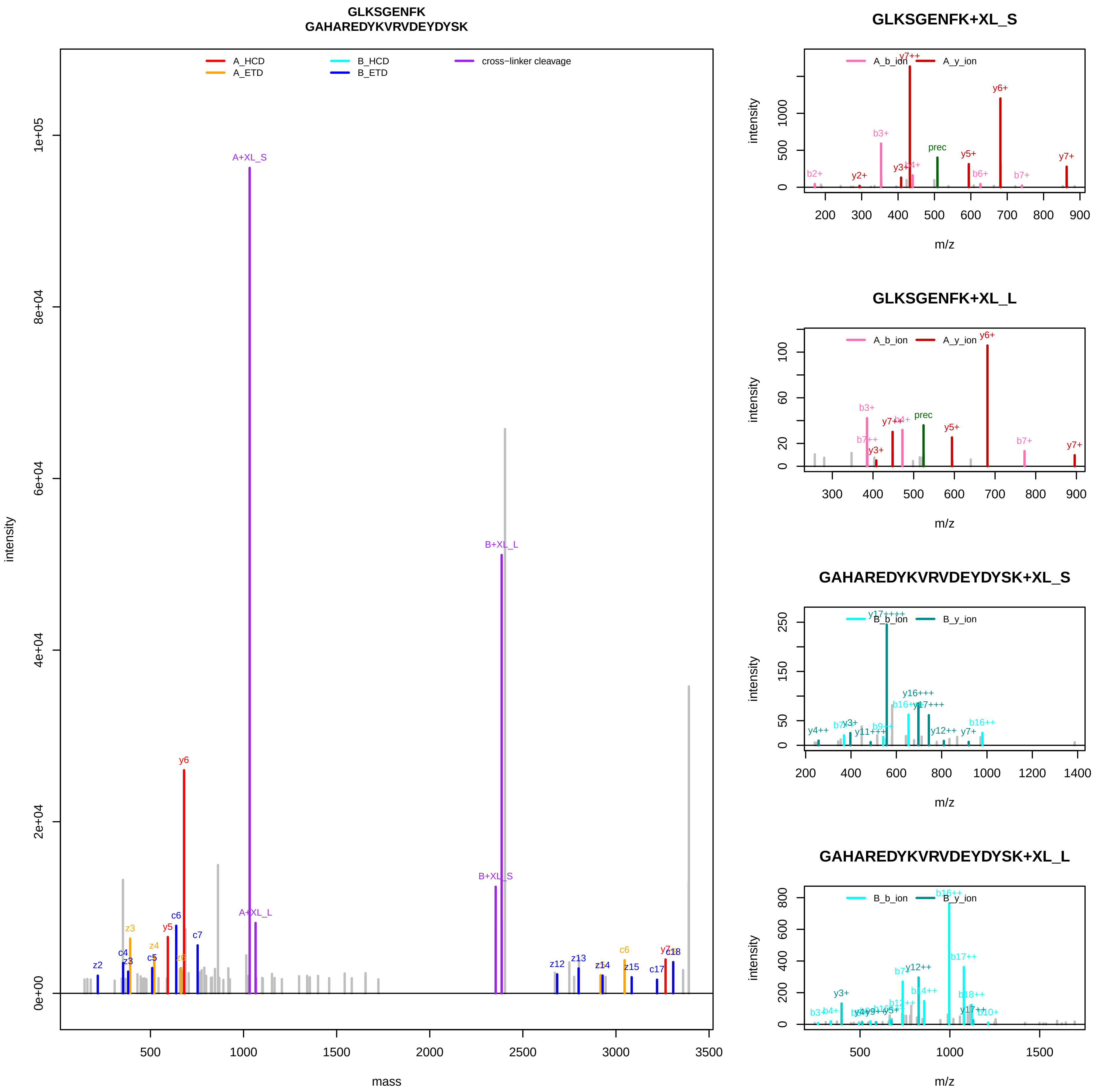

GLKSGENFK
GAHAREDYKVRVDEYDYSK
A_HCD
A_ETD
B_HCD
B_ETD
cross−linker cleavage
A+XL_S
B+XL_L
B+XL_S
A+XL_L
intensity
mass
0e+00
2e+04
4e+04
6e+04
8e+04
1e+05
500
1000
1500
2000
2500
3000
3500
y6
y5
c6
c7
z2
c4
z3
c5
z4
z6
z12
z13
z14
z15
c6
c17
c18
y7
GLKSGENFK+XL_S
A_b_ion
A_y_ion
y7++
y6+
b3+
prec
y5+
y3+
b4+
b2+
y2+
b6+
b7+
y7+
m/z
GLKSGENFK+XL_L
b7++
GAHAREDYKVRVDEYDYSK+XL_S
B_b_ion
B_y_ion
y17++++
y16+++
b16+++
y17+++
y4++
b7+
y11+++
b9++
y12++
b16++
GAHAREDYKVRVDEYDYSK+XL_L
b16++
b17++
b7+
y12++
b14++
b18++
y3+
b3+
b4+
y17++
b10+
